# *Dermatophilus congolensis* associated bronchopneumonia in an alpaca

**DOI:** 10.1002/vms3.213

**Published:** 2019-11-14

**Authors:** Neil U. Horadagoda, Sara Biasutti, Marina Gimeno, Andrew Dart

**Affiliations:** ^1^ University Veterinary Teaching Hospital Camden University of Sydney Camden NSW Australia

**Keywords:** bacteria, bronchopneumonia, Dermatophilus, granulomatous, South American camelid, Splendore–Hoeppli

## Abstract

A severe, chronic, locally extensive granulomatous bronchopneumonia was diagnosed on post‐mortem and histopathological examination of an adult alpaca. *Dermatophilus congolensis* organisms were isolated from the lungs and genotypic identification of aerobic culture was confirmed by sequence analysis of the entire 16S rDNA gene. This is the first report of *D. congolensis*‐associated bronchopneumonia in any species.

## Graphical abstract

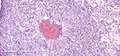

## 1 INTRODUCTION

*Dermatophilus congolensis* is a Gram‐positive filamentous bacterium which is usually known to cause skin disease in a wide range of domestic and wild animals throughout the world, especially in the humid tropics and subtropics. The dermal lesions are characterised by raised, crusty, alopecic and sometimes papillomatous lesions covered by thick keratinaceous incrustations (Hargis & Myers, 2017; Mauldin & Peters‐Kennedy, 2015). In temperate countries, sheep and goats are commonly affected and a sporadic proliferative pyogranulomatous pododermatitis has been seen in alpacas in Australia (Horadagoda unpublished data). Apart from skin infections, in rare instances, the organism can cause pyogranulomatous infections in the lymph nodes (Byrne, Rand, McElliott, Samitz, & Brault, 2010). To the author's knowledge, this is the first report of *D. congolensis* associated with a case of bronchopneumonia in an animal.

## 2 CASE HISTORY

A 12‐month‐old male alpaca was presented to the University of Sydney Veterinary Teaching Hospital, Camden for evaluation of weight loss and cough of 2 weeks duration. The alpaca had been treated 6 months previously for draining of abscesses under the jaw and in the groin, thought to be caseous lymphadenitis (CLA). These abscesses had resolved, and the alpaca had been otherwise clinically normal and had a good appetite until 2 weeks prior to presentation.

The animal was quiet, alert and responsive on presentation. On physical examination the mucous membranes were slightly pale, respiratory rate was elevated (60 bpm; normal 10–30 bpm) with increased abdominal effort and nostril flaring. There were diminished bilaterally equivalent lung sounds ventrally but crackling and pleural rub sounds were heard dorsally on auscultation, consistent with a pleural effusion and pneumonia. A venous blood sample showed a moderate anaemia (red cell count: 4.87 × 10^12^/L, reference value 10.5–17.1; haemoglobin: 67 g/L, reference value 117–191; packed cell volume 0.15 L/L, reference value 0.27–0.45) with a mild leucocytosis (24.3 × 10^9^/L, reference value 7.9–23.6). The leucocyte changes were characterised by a neutropenia (2.19 × 10^9^/L, reference value 4.6–16.1) and a marked increase in band neutrophils (9.23 × 10^9^/L, reference value 0–0.2) reflecting a degenerative left shift and a marked eosinophilia (10.45 × 10^9^/L, reference value 0–4.2). The significant biochemical findings included a marked hypoalbuminaemia (19 g/L, reference value 31–52) and a mild hyperglobulinaemia (43 g/L, reference value 26–40). A standing lateral thoracic radiograph revealed heavy alveolar infiltrates throughout the cranial and ventral thorax with marked soft tissue opacity effacing the margins of the cardiac silhouette/ventral diaphragm and multiple air bronchograms. Severe bronchopneumonia with or without pleural effusion was considered likely. Given the clinical signs, blood sample and radiographic findings, the owners were offered a very guarded prognosis for survival so elected to euthanise the alpaca.

**Figure 1:**
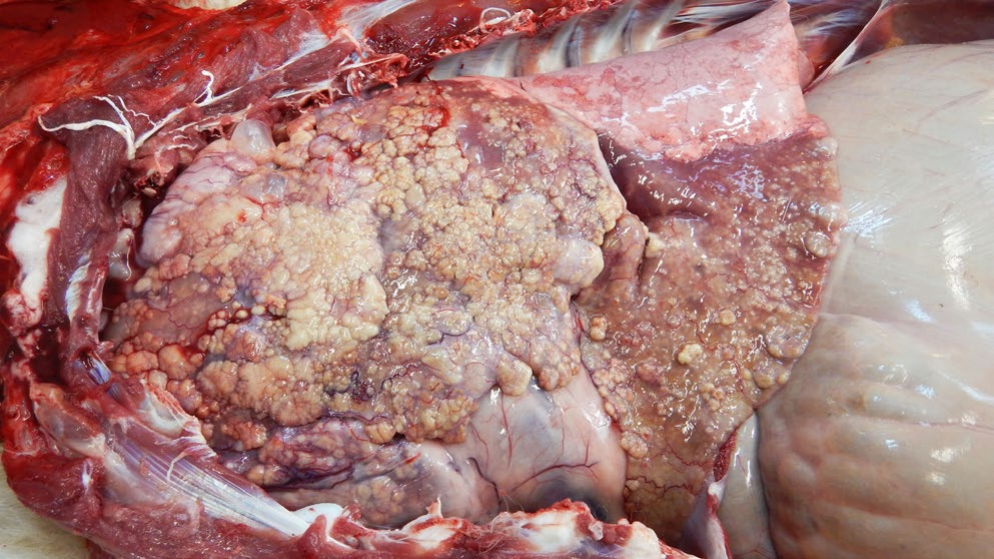
Left lateral view of the lungs showing 60%–70% consolidation of the cranioventral  lobes with multifocal to coalescing, nodules on the pleural surface

Post‐mortem examination revealed marked, locally extensive, cranioventral consolidation involving 60%–70% of the lungs with the pleural surfaces containing multifocal to coalescing, firm creamy white nodules (Figure 1). On the cut surface, the nodules extended into the lung parenchyma and there were multiple fibrous adhesions between pleural surfaces. The tracheobronchial lymph nodes were enlarged. The left inguinal lymph node was firm and enlarged. All other body systems were grossly normal.

**Figure 2:**
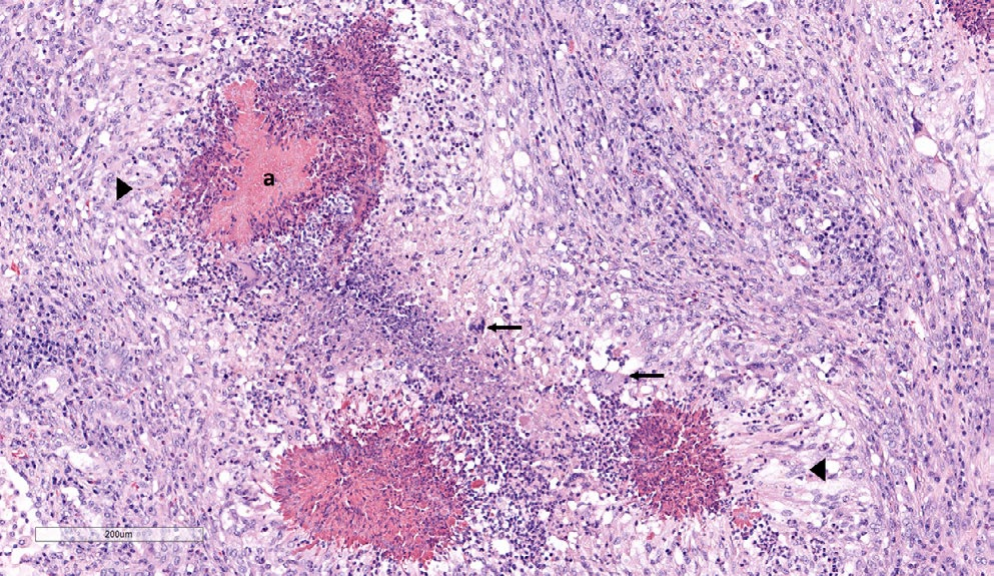
Lungs with multifocal pyogranulomatous inflammatory foci with epithelioid cells (arrow heads) and multinucleated giant cells (arrows) surrounding Splendore–Hoeppli material (a) H&E stain; ×100)

**Figure 3:**
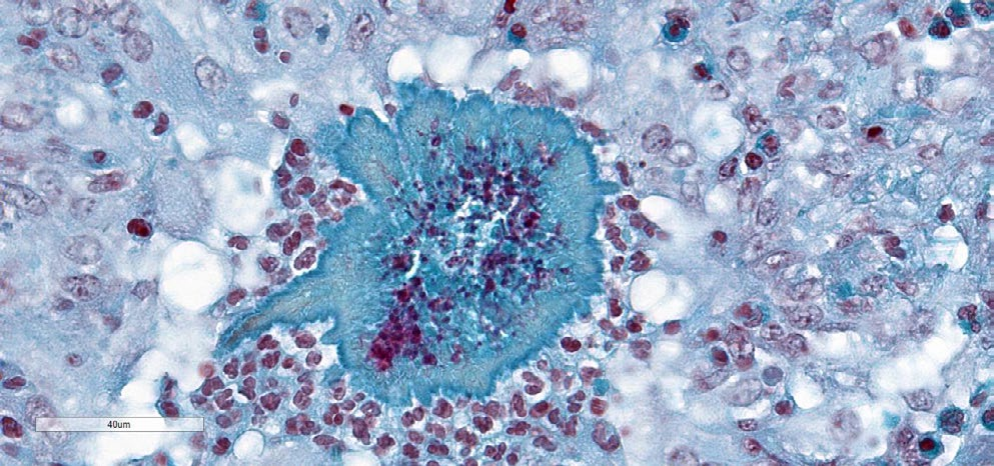
Gram‐positive filamentous organisms surrounded by pale blue staining Splendore–Hoeppli material (Gram stain; ×600)

Histopathology of the lung revealed multifocal, expansile inflammatory foci comprising of a central core of necrotic debris interspersed by degenerate neutrophils surrounded by macrophages, epithelioid macrophages and occasional multinucleated giant cells (Figure 2). Peripheral to these inflammatory foci were a capsule of fibrous tissue admixed with lymphocytes, plasma cells and moderate numbers of eosinophils. In the centre of some of the inflammatory foci, there were filamentous Gram‐positive organisms surrounded by eosinophilic proteinaceous aggregates of Splendore–Hoeppli material (Figure 3). The eosinophilic aggregates were negative for amyloid and fibrin on Congo red and phosphotungstic acid haematoxylin staining respectively. In the lung tissue surrounding the inflammatory foci, the alveolar spaces contained homogenous pink material interspersed by macrophages, neutrophils and large numbers of eosinophils.

The nodular masses on the pleural surface of the lungs also contained multiple, granulomatous inflammatory foci with central aggregates of Splendore–Hoeppli material as described within the lung parenchyma. The architecture of the inguinal lymph node was completely effaced on histopathology by multifocal to coalescing foci of necrosis and mineralisation interspersed by neutrophils and fibroplasia. Additionally, there were multiple foci of granulomatous inflammation as described for the lungs with central aggregates of Splendore–Hoeppli material.

**Figure 4:**
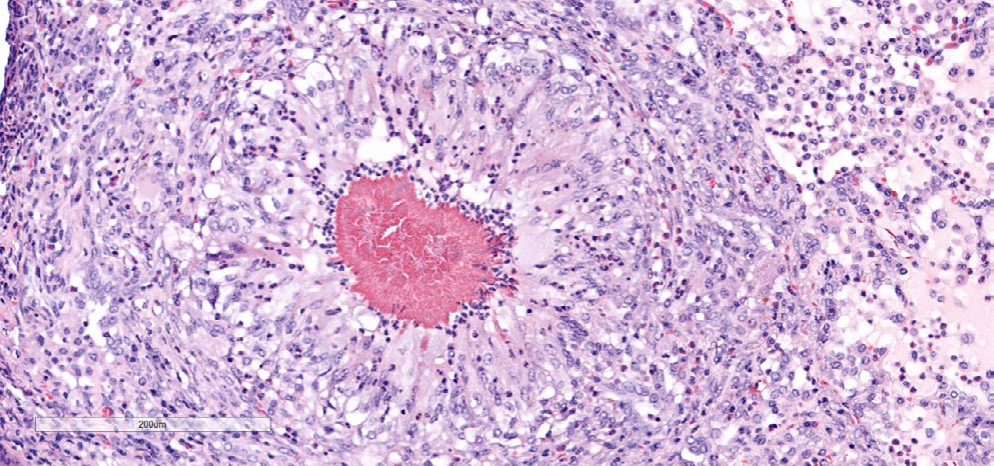
Image of the organism and tissue to be considered for online cover and blog publication

Aerobic culture of the lung tissue yielded a slow growing (48–72 hr), Gram‐positive filamentous organism (Figure 4). Genotypic identification of culture was made by sequence analysis of the entire 16S rDNA gene. The sequence obtained was 99.7% identical to the type strain of *D. congolensis* NCTC 13039 (NCTC LT906453) which is equivalent to type strain DSM 44180^T^ (Kagia & Liu, 2014).

## 3 DISCUSSION

In its natural habitat, *D. congolensis* is thought to maintain itself in small foci of infection on carrier animals or within scab particles in dusts where it can survive up to 3 years (Markey, Leonard, Archambault, Cullinane, & Maguire, 2013). The presence of a cranioventral distribution of pneumonic consolidation in the lungs of this alpaca suggests a bronchogenic route of infection probably through inhalation of the organism in infected dust particles. This proposition is further supported by the absence of gross lesions in the upper respiratory tract which indicates that the pulmonary infection here is not an extension from an already established inflammatory focus within the respiratory system.

A distinctive histomorphological feature noted in the lungs and lymph nodes of this alpaca were the foci of granulomatous inflammation often with centrally located individual or bacterial colonies surrounded by radiating, homogenous eosinophilic substance known as Splendore–Hoeppli material. This material is thought to be antigen–antibody complexes together with tissue debris and fibrin (Hargis & Myers, 2017). Splendore–Hoeppli material is seen with certain persistent, poorly degradable, foreign body antigens and with fungal infections such as coccidioidomycosis, sporotrichosis as well bacterial diseases caused by *Actinomyces bovis, Actinobacillus lignieresii and Staphylococcus aureus* (Hargis & Myers, 2017; Jones, Hunt, & King, 1997). To the author's knowledge, the Splendore–Hoeppli phenomenon is not reported as a histomorphological feature in infections caused by *D. congolensis.*

The histology of the inguinal lymph node showed a concurrent infection with granulomatous inflammation characteristic of *D. congolensis* abutting large areas of necrosis with mineralisation, consistent with CLA caused by *Corynebacterium pseudotuberculosis.* Concurrent infections of dermatophilosis and CLA has been reported in outbreak forms in camels (Tarazi & Al‐Ani, 2016). In the present case, this lesion may reflect residual infection from the previously, suspected episode of CLA, 6 months prior to this presentation. According to Valli, Kiupel, Bienzle, and Wood (2015) as a general rule, once the infection is established in a node it is persistent although in some instances it may be cleared when the node ruptures. The treatment records of the earlier presentation at this Hospital reported that the lesion content was shelled out from the submandibular and superficial inguinal lymph nodes at surgery and the wounds left to heal without sutures at the site of incision. This procedure is likely to have some residual organisms which are responsible for the histological changes in the inguinal lymph node.

In the blood work, the marked eosinophilia is a significant finding, and this is reflected by the mobilisation of circulatory eosinophils by pulmonary granulomatous inflammatory foci containing Splendore–Hoeppli material and the surrounding pneumonic lung tissue. Although the functions of eosinophils are not fully understood they are known to be involved in the modulation of allergic inflammation and immune‐complex reactions (Weiser, 2012). Given that Splendore–Hoeppli material originates from antigen–antibody complexes, it is likely that eosinophils are attracted to this material resulting in an increased demand in circulation and activation of the haemopoietic system. The association between Splendore–Hoeppli material and eosinophils is supported by another report which describes multiple eosinophilic granuloma in a nasal lesion caused by toxigenic *Corynebacterium ulcerans* although the occurrence of a concurrent eosinophilia is not mentioned (Murakami et al., 2014).

*Dermatophilus congolensis* is generally considered a dermal pathogen affecting a wide range of animals with rare occurrence of lesions in other tissues. The clinical findings reported in this paper have for the first time shown that *D. congolensis* can be associated with bronchopneumonia and that the organism should be considered as a pathogen in investigations relating to chronic pulmonary infections characterised by granulomatous inflammation. The presence of the organism in carrier animals and within dust particles for long periods in the environment could potentially allow dissemination of the pulmonary disease or the establishment of new infections across different species.

## CONFLICT OF INTEREST

The authors have no conflict of interest.

## ETHICAL STATEMENT

The authors confirm that the ethical policies of the journal, as noted on the journal's author guidelines page, have been adhered to. No ethical approval was required as this is an investigation of an animal at post‐mortem examination.
